# RDI Calculator: An Analysis Tool to Assess RNA Distributions in Cells

**DOI:** 10.1038/s41598-019-44783-2

**Published:** 2019-06-04

**Authors:** Michael Stueland, Tianhong Wang, Hye Yoon Park, Stavroula Mili

**Affiliations:** 1 Laboratory of Cellular and Molecular Biology, Center for Cancer Research, National Cancer Institute, NIH, Bethesda, MD USA; 2 Department of Physics and Astronomy, Seoul National University, Seoul, Korea

**Keywords:** Fluorescence in situ hybridization, Molecular imaging, RNA transport, Software

## Abstract

Localization of RNAs to various subcellular destinations has emerged as a widely used mechanism that regulates a large proportion of transcripts in polarized cells. A number of methodologies have been developed that allow detection and imaging of RNAs at single-molecule resolution. However, methodologies to quantitatively describe RNA distributions are limited. Such approaches usually rely on the identification of cytoplasmic and nuclear boundaries which are used as reference points. Here, we describe an automated, interactive image analysis program that facilitates the accurate generation of cellular outlines from single cells and the subsequent calculation of metrics that quantify how a population of RNA molecules is distributed in the cell cytoplasm. We apply this analysis to mRNAs in mouse and human cells to demonstrate how these metrics can highlight differences in the distribution patterns of distinct RNA species. We further discuss considerations for the practical use of this tool. This program provides a way to facilitate and expedite the analysis of subcellular RNA localization for mechanistic and functional studies.

## Introduction

RNA molecules are transcribed in the nucleus and exported to the cytoplasm where they usually serve as messengers for decoding the genetic information into protein products. In the cytoplasm, RNAs are either uniformly distributed or they can become localized to various subcellular destinations through mechanisms including motor-based active transport or diffusion followed by anchoring^1,2^. It has been increasingly appreciated that a large proportion of the mammalian transcriptome becomes differentially distributed in the cytoplasm through such mechanisms^3–7^. Hundreds to thousands of RNAs are localized in axons or dendrites of neurons^8–10^, apical or basal surfaces of epithelial cells^11,12^, or the leading edge and protrusive regions of mesenchymal migrating cells^13–15^. Importantly, changes in RNA localization are linked to physiological responses and have functional consequences^14,16–19^, highlighting the importance of understanding and exploiting the underlying mechanisms.

While a number of methods are available for high-resolution, single-molecule imaging of RNAs either in fixed or live cells^20,21^, relatively fewer options exist for describing the observed RNA distributions in an unbiased, objective manner and in quantifiable terms. In highly polarized cells, such as neurons, quantitative assessment of RNA localization from imaging data can be done by assigning RNAs into easily distinguishable compartments such as dendrites, axonal shafts or growth cones^8,22^. Such distinctions become much harder and inherently biased in smaller cell types which have either irregular and varied morphologies or lack clear boundaries of functional locations. For example, there is no objective way of defining the exact spatial boundaries of the leading edge of a migrating cell or the apical and basal surfaces of an epithelial cell^11,12,16,23^. Furthermore, such demarcations apply a binary choice onto the description of RNA distributions, thus precluding the ability to detect and differentiate distributions which result from more gradual RNA concentration gradients.

To address these issues, various quantitative metrics have been used to describe cytoplasmic RNA distributions^14,24–26^. These approaches identify RNAs within a specified cell boundary and usually describe their spatial distribution in relation to particular cellular features such as the plasma membrane, the nuclear envelope or the cell centroid. It is therefore important that these features are accurately identified during image analysis. One case in point concerns RNAs targeted to peripheral protrusive regions of mesenchymal migrating cells^13,14^. Such protrusions are thin and because they contain a relatively small cytoplasmic volume are less efficiently detected by fluorescent stains commonly used to demark the cell volume. Failure to include them during the identification of the cell boundary could lead to omission of RNAs and thus inaccurate calculation of distribution metrics. Automated cell segmentation methods might not always accurately identify all relevant features, especially in images with low signal-to-noise ratios.

We report here an interactive analysis tool (named RDI Calculator, for RNA Distribution Index Calculator) that automatically identifies cellular and nuclear boundaries, but additionally allows their easy editing and correction when necessary. The program then calculates different RNA distribution metrics from single cells, thus facilitating an accurate, unbiased analysis of large sample sizes. We employ this program to demonstrate how this analysis can quantitatively highlight differences in the distribution patterns of various endogenous RNAs. We further present practical considerations for the implementation of this tool.

## Materials and Methods

### Cell culture

NIH/3T3 mouse fibroblast cells (ATCC) were grown in DMEM supplemented with 10% calf serum, sodium pyruvate and penicillin/streptomycin (Invitrogen) at 37 °C, 5% CO_2_. MDA-MB-231 breast cancer cells (ATCC) were grown in Leibovitz’s L15 media supplemented with 10% fetal bovine serum and penicillin/streptomycin at 37 °C in atmospheric air. Cell lines have been tested yearly for mycoplasma and are free of contamination.

### RNA *in situ* hybridization

For *in situ* hybridization, cells were plated on fibronectin-coated polyacrylamide gels, or on fibronectin-coated 20 μm micropatterned line tracks (CYTOOchips Motility, CYTOO), or on fibronectin-coated glass coverslips for 2–3 hours and subsequently fixed in 4% paraformaldehyde for ten minutes. FISH was performed with QuantiGene ViewRNA ISH Cell Assay kit (Affymetrix, cat# QVCM0001) according to the manufacturer’s instructions. The Affymetrix probe sets used were: Ddr2 cat# VB1-14375, RhoA cat# VB6-14572, Cyb5r3 VB1-18647, Arpc3 cat# VB6-14571. To detect PolyA RNAs, LNA modified oligodT probes (30 nucleotides) labeled with ATTO-655 were added during hybridization, pre-amplification, amplification and last hybridization steps of QuantiGene ViewRNA ISH Cell Assay. Nuclei were stained using DAPI and cells were additionally stained with Cell mask stain (Thermo Fisher Scientific) to obtain cell outlines.

### Preparation of polyacrylamide gel substrates

Thin polyacrylamide gels were prepared on glass coverslips as described previously^14^. Gels were coated with 0.1 mg ml^−1^ fibronectin (Sigma-Aldrich) overnight.

### Image acquisition

Images were obtained using a Leica SP8 or a Zeiss 780 confocal microscope (equipped with an HC PL APO 63x oil CS2 objective, 1.40 NA or a HC PL APO 40x oil CS2 objective, 1.30 NA). Z-stacks through the cell volume were obtained and files were analyzed using RDI Calculator as detailed below.

### Statistics

Prism 7 by GraphPad software was used for graph generation and statistical analyses. For multiple comparisons, one-way ANOVA was used with Tukey’s multiple comparisons test. For comparison of two data sets, Student’s t-test was used. Significance level was set to p < 0.05.

## Automated Analysis Algorithm

### Overview and image requirements

**Figure 1:**
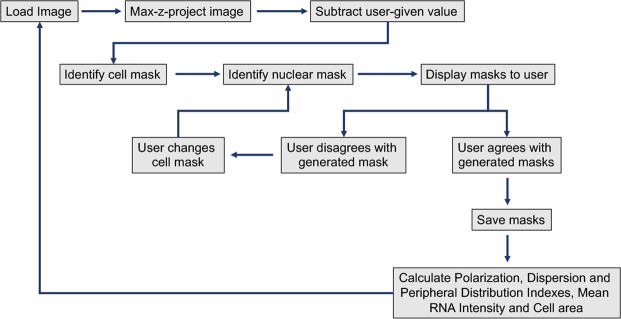
Flowchart of automated analysis steps. Chart detailing the steps taken by the program and the points of user input (see text for details).

The input files are 4-channel images and their format can be either.lif or.tif. The images can be either confocal z-stacks or single-plane images. If stacks are provided, the program will generate a maximum intensity, z-projected file. The subsequent calculations require first determination of the outer boundaries of the cell being analyzed as well as of the position of the nucleus within the cell being analyzed. Therefore, usually, one channel is used to acquire an image of the cell nucleus (for example through DAPI staining) and a second channel is used to acquire an image of the whole cell volume (for example through staining with appropriate cell stain reagents or through immunostaining, or fluorescence imaging, of diffuse cytoplasmic proteins). The additional two channels are used for imaging of the RNAs to be analyzed through single molecule *in situ* hybridization (see methods). Binary masks of the whole cell and nucleus are subsequently generated and displayed for user-approval. If accepted, the binary mask files are automatically named and saved, otherwise a number of options are presented for manual editing of individual masks. RNA distribution metrics (PDI, PI, DI), mean RNA intensity and cell area are then calculated and the results are exported and saved in a .csv spreadsheet format (Fig. 1). Step-by-step instructions for running the analysis are detailed in Supplementary file 1. Supplementary source files include test images and scripts for running the analysis.

Images can be any bit-depth but 12-bit or higher are recommended. As usual for images used for quantitative signal analysis, using the full-dynamic range of the detector and acquiring non-saturated images is important. This program has been generated and tested in MATLAB 9.1 and later versions and runs in both Windows and Mac operating systems.

### Cell and nucleus identification

To generate a binary mask of the cell area, images of the whole cell volume are acquired, aiming for good contrast between the cell and the background. A Sobel operator is then applied to find the edges of the cell. Specifically, a 3 × 3 filter is applied across the image in both X and Y dimensions to measure the gradient of signal intensity. A threshold is then applied to this gradient, resulting in a binary image highlighting the boundaries of the cell. Next, a slight dilation (of one pixel in all directions) is applied to the binary image. A flood-fill operation is further applied to produce a filled-in binary mask of the cell. Note that the algorithm recognizes the cell that covers the largest area in an image. For images with multiple cells, individual cells could be cropped out, or alternatively different cells could be selected by rejecting the automatically selected cell and redefining the cell mask with the available editing options (see below).

To generate a binary mask of the nuclear area, nuclear images (e.g. through DAPI staining) are acquired, aiming again for a good contrast between the nucleus and background. First, a Wiener filter is applied to the nuclear channel. Specifically, across the image, variance is computed in a 5 × 5 pixel area. Pixel intensity is then smoothed out in inverse relationship to variance. This preserves the edges in the image, while eliminating random background noise. Next, an automatic Otsu threshold is applied to the image, producing a binary image as a result. This binary image of the nucleus is then multiplied by the binary image of the cell mask, which removes any nuclear signal not inside the cell mask, thus eliminating any nuclei in the original image that are not within the cell being analyzed. Finally, a flood-fill operation in applied, producing a nuclear mask for only the cell of interest.

### User input on cell and nuclear mask identification

The outlines of the cell and nuclear masks, which are generated through the above-mentioned process, are then displayed and overlaid with the corresponding image channels or with the RNA channels, so that the user can assess whether they are suitable for use in downstream analysis. For images with adequate signal-to-noise, these steps are usually sufficient, and the generated masks can be automatically saved. However, in certain cases the standard method of identifying the boundaries of the cell may not be sufficient. For these scenarios, a number of methods have been built in to allow the user to modify the image, modify the Sobel edge detection method, or modify the generated mask itself.

#### Wiener noise removal

This allows the user to pre-process the cell mask channel prior to the Sobel edge detection. As mentioned above, this uses a 5 × 5 filter to detect signal variance across the image, and smooth out pixel intensity in areas of relatively low variance. This can be repeated any number of times to increase the noise-removal effect, though for most purposes once should be adequate.

#### Image sharpening

This allows the user to pre-process the cell mask channel, prior to the Sobel edge detection, with unsharp masking. This increases the contrast at the edges of signal and non-signal. This may help if the cell staining is faint, especially at cell peripheries. This can also be iterated multiple times.

#### Dilation

This allows the user to dilate the generated mask by one pixel in all directions. This can be done multiple times for increased dilation. This function can be useful in ensuring that RNAs found at peripheral edges of the cell are included in the masked area used for analysis.

#### Threshold for edge detection

This allows the user to set the threshold for the Sobel Edge detection method. Specifically, the gradient of signal intensity for each pixel is given a value. A potential threshold is automatically calculated, and the user can multiply that by a given number. The default for this is 0.5, with lower numbers detecting more potential edges and larger numbers being more stringent.

#### Using a polygon tool to alter the cell mask

The following sections denote ways that the user can manually draw a polygon to alter the cell mask. The user has the option to draw a polygon on top of an overlay of the original cell mask channel, and this is then used to modify the calculated cell mask boundaries. Calculation of the boundaries of the nuclear mask is done after this step, so if a new region is highlighted with nuclear signal, the nuclear mask may change.

Draw: In this option, the user draws a polygon to approximate the boundaries of the cell. This will entirely replace the original cell mask with the drawn polygon.

Limit: In this option, the user draws a rough polygon only around the region they want analyzed. This will multiply the polygon by the calculated cell mask, so that anything not circled by the polygon will be excluded.

Expand: In this option, the user draws a polygon connecting to the generated cell mask, around any additional area to be analyzed. This will add the polygon to the generated mask.

### Output metrics

Calculations of RNA distribution indexes are performed using maximum-intensity projections of z-stacked images. Intensity values, above a user-determined threshold, are used for analysis. The program does not attempt to identify discrete RNA particles or assign numbers of RNA molecules contained within clusters or diffraction-limited RNA spots. Instead, pixel intensities are used in order to accommodate analysis in cases where single RNA identification cannot be confidently performed, such as in the case of abundant RNA species, or when imaging polyadenylated RNA. We note that we have compared intensity-based measurements with measurements after single RNA spot detection and found the calculated metrics to be very similar.

**Figure 2:**
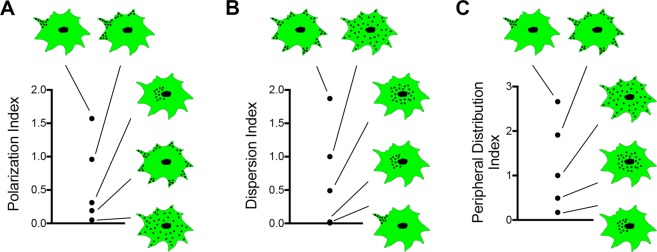
RDI-derived metrics on simulated images. Images simulating various potential RNA distributions were analyzed using the RDI calculator program and the derived values were plotted. (**A**) Polarization index increases with increased polarization of the RNA signal and with increased distance from the cell centroid. (**B**) Dispersion index is 1 for a diffuse RNA. Concentration of the RNA in any region is characterized by a DI index less than 1 and is inversely correlated with the degree of RNA concentration. An RNA that is distributed towards the cell periphery exhibits a DI value larger than 1, but this is affected by the degree of polarization. (**C**) The peripheral distribution index is also 1 for a diffuse RNA. This index is proportional to the distance from the nucleus and is not affect by the polarization of the RNA distribution.

Three different indexes are calculated, a polarization index, a dispersion index and a peripheral distribution index. Each index describes a distinct aspect of how a population of RNA molecules are distributed within the cell cytoplasm^14,24^. The Polarization Index (PI) is calculated by identifying the centroid of the RNA signal and measuring its displacement from the centroid of the cell. This displacement is divided by the radius of gyration, calculated as the root-mean-square distance of all pixels to the centroid of the cell, in order to normalize the polarization to the size and elongation of the cell^24^.

$$PI=\frac{\sqrt{{({\bar{x}}_{RNA}-{\bar{x}}_{cell})}^{2}+{({\bar{y}}_{RNA}-{\bar{y}}_{cell})}^{2}}}{R{g}_{cell}},$$

where ${\bar{x}}_{RNA}$, ${\bar{y}}_{RNA}$ are the coordinates of the RNA centroid and ${\bar{x}}_{cell}$, ${\bar{y}}_{cell}$, are the coordinates of the centroid of the cell in the two dimensional image, and *Rg*_*cell*_ is the radius of gyration. PI values increase with increased polarization of the RNA signal and with increased distance from the cell centroid (Fig. 2A).

To derive the Dispersion Index (DI), the second moment of RNA pixel intensity positions relative to the centroid of the total RNA signal is calculated.

$${\mu }_{2}=\,\sum _{i,j}{r}_{ij}^{2}\frac{{I}_{ij}}{{\sum }_{i,j}{I}_{ij}},$$

where *r*_*ij*_ is the distance of the pixel (i,j) to the centroid of the RNA signal and *I*_*ij*_ is the intensity value of pixel (i,j) in the two dimensional image. To normalize for differences in cell morphology, the second moment of the RNA is divided by the second moment of a hypothetical uniform distribution, which is derived as the second moment of all pixels within the binary cell mask image^24^. A completely diffuse RNA has a DI value of 1. The DI value of an RNA that is concentrated in any region within the cell is less than 1 and is inversely correlated with the degree of RNA concentration. An RNA that is distributed towards the cell periphery exhibits a DI value larger than 1, but this is affected by the degree of polarization (Fig. 2B).

The Peripheral Distribution Index (PDI) is calculated similar to the dispersion index, but in this case the second moment of RNA pixel intensity positions is calculated relative to the centroid of the nucleus^14^. This metric is not affected by the polarization of the RNA distribution. PDI value is 1 for a completely diffuse RNA, it is less than 1 for a perinuclear RNA and more than 1 for a peripherally distributed RNA (Fig. 2C).

In addition to the RNA distribution indexes described above, the program also reports the cellular area, in μm^2^ units, utilizing the pixel dimension information from the metadata of the provided images, as well as the average RNA signal within the cell being analyzed. For this, each RNA channel is multiplied by the cell mask. This multiplies all RNA signal outside the cell by 0, and all signal inside the cell by 1. Every pixel of the RNA channel is then summed and divided by the pixel sum of the cell mask. This is the total RNA signal divided by cell area, or the average RNA signal across the area of the cell.

## Results

### RDI analysis can quantitatively differentiate distinct RNA distributions in cell populations

**Figure 3:**
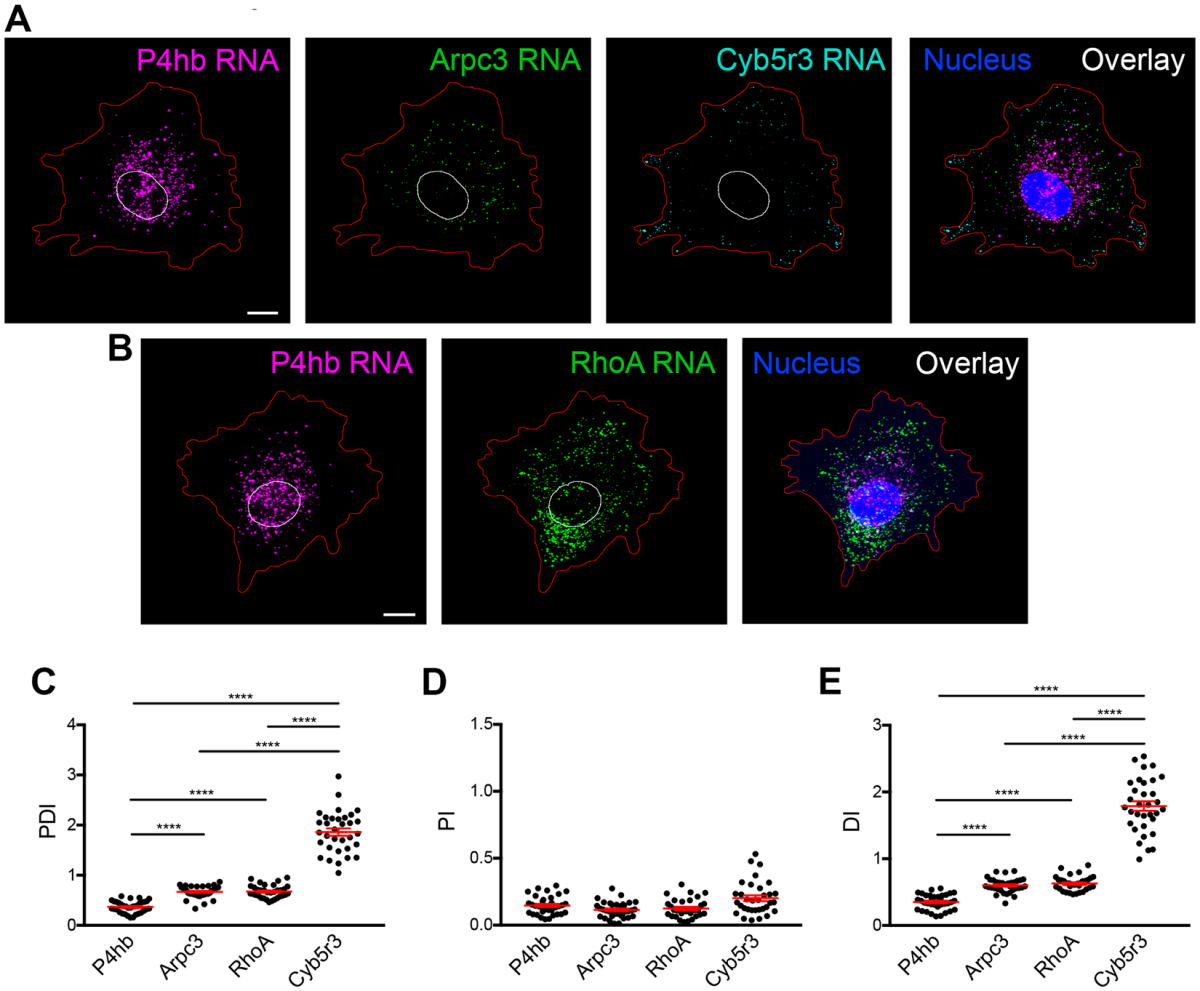
RDI analysis quantitatively differentiates RNA distributions in cell populations. (**A**,**B**) Mouse fibroblast cells were processed by *in situ* hybridization to detect the indicated RNAs. Red line: cell outline. White line: nuclear outline. (**C**–**E**) Images from multiple cells were analyzed using the RDI calculator and computed PDI (**C**), PI (**D**) and DI (**E**) values were plotted. N = 33–35 cells. Similar results were observed in more than 3 independent experiments. ****p-value < 0.0001 by one-way analysis of variance with Tukey’s multiple comparisons test. Scale bars: 10 μm.

To illustrate how this analysis can be used to describe the distribution of distinct RNA species, we used single molecule *in situ* hybridization to detect mRNAs in mouse fibroblast cells. We visualized RNAs that exhibit distinct distribution patterns (Fig. 3A,B). Specifically, these include: (1) the P4hb mRNA, encoding the beta subunit of prolyl-4-hydroxylase, which functions as an ER chaperone^27^, (2) the Arpc3 mRNA, encoding subunit 3 of the actin-nucleating Arp2/3 complex^28^, (3) the RhoA mRNA, encoding the RhoA GTPase involved in organization of the actin cytoskeleton^29^, and (4) the Cyb5r3 mRNA, encoding cytochrome b5 reductase 3 involved in fatty acid and cholesterol metabolism^30^. These RNAs exhibit visibly distinct patterns of distribution in the cytoplasm (Fig. 3). Specifically, the P4hb RNA exhibits a perinuclear distribution and does not reach to the cell periphery, likely reflecting the ER association of its encoded protein. The Arpc3 RNA appears diffuse, exhibiting a more uniform distribution throughout the cell body. Nevertheless, it is not prominently found within peripheral protrusive regions. The RhoA RNA is diffusely distributed similar to Arpc3 RNA. The Cyb5r3 RNA exhibits a peripheral distribution being relatively absent from the perinuclear region and concentrating within peripheral protrusions (Fig. 3A,B).

Using the RDI calculator, PDI, PI and DI values were derived for each of the above RNAs from multiple individual cells (Fig. 3C–E). The P4hb RNA has the lowest PDI value (with a mean value of 0.36), reflecting its perinuclear distribution. This value is significantly different from the PDI values of Arpc3 or RhoA RNAs. Both Arpc3 and RhoA RNAs have similar PDI indexes (mean values of 0.66 for Arpc3 and 0.67 for RhoA), consistent with their indistinguishable distribution. Furthermore, indicating the absence of both of these RNAs from peripheral protrusions, the PDI index for both is lower than 1. The Cyb5r3 RNA has the highest PDI index (mean value of 1.86) reflecting its pronounced accumulation at peripheral protrusive regions (Fig. 3C). Therefore, differentially distributed RNAs can be robustly distinguished using the PDI metric.

### Relationship between DI and PDI values varies depending on the context

As shown in Fig. 3C,E, DI values for the tested RNAs parallel the PDI values and reveal the same differences in distribution. This concordance in PDI and DI values is predicted and observed also in the simulated images (Fig. 2B,C). This concordance is expected when RNA distributions are non-polarized. Indeed, the cells analyzed in Fig. 3 are spreading briefly on uniformly fibronectin-coated surfaces and thus do not exhibit any obvious morphological or functional polarization. Consistently, RNA distributions are non-polarized, reflected in the low PI values (ranging from 0.12-0.24) (Fig. 3D). Under such conditions, there is largely agreement between the DI and PDI metrics.

**Figure 4:**
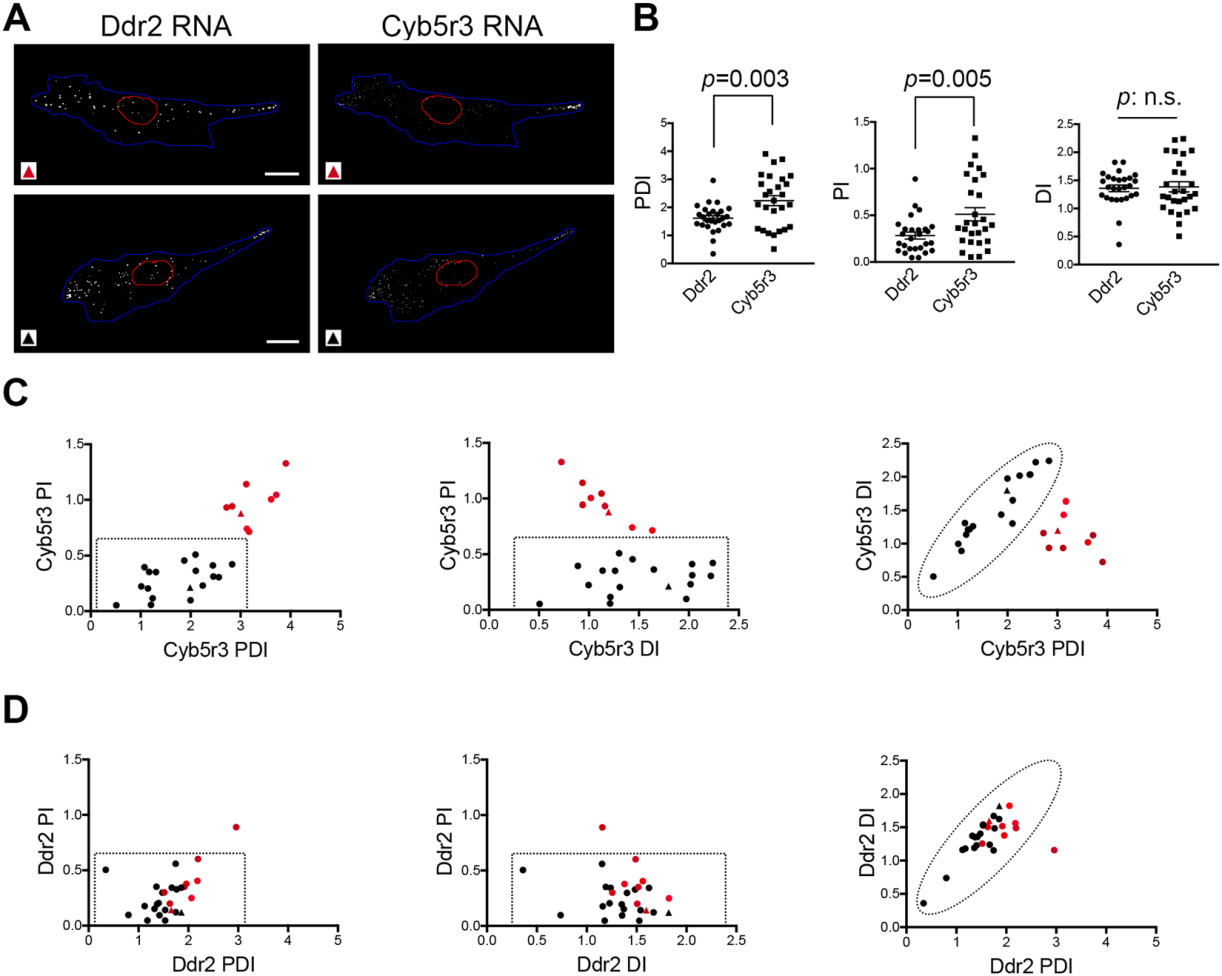
Relationships between the three metrics can distinguish subsets of cells in a population. **(A**) Cells plated on 20 μm-line micro-patterned substrates were processed by *in situ* hybridization to detect the indicated RNAs. Red and black triangles indicate the cells that correspond to the data points in (**C**,**D**) indicated respectively by red and black triangles. Scale bars: 15 μm. (**B**) Images of multiple cells were analyzed using the RDI calculator and PDI, PI and DI values were plotted. p-values by Student’s t-test. N = 27 cells. Similar results were observed in two independent experiments. (**C**,**D**) Scatter plots of pairwise correlations of values from (**B**). Dotted lines encompass the range of values exhibited by the Ddr2 RNA in all, but one, cells of the population.

By contrast, if RNA distributions are polarized, DI and PDI metrics are expected to diverge and to be increasingly different with increasing distance of the RNAs from the nucleus (Fig. 2B,C). To illustrate this point, we imaged mouse fibroblast cells migrating on micropatterned substrates consisting of 20 μm-wide lines. On these defined line-patterns, a large proportion of the cells polarize to form a leading edge and a trailing, retracting tail (Fig. 4A). We visualized, in these cells, the Cyb5r3 RNA as well as the Ddr2 RNA, encoding a collagen receptor, and performed RDI analysis from multiple images (Fig. 4). Both RNAs are peripherally localized and belong to a co-regulated group that depends on the APC (Adenomatous Polyposis Coli) protein for their peripheral localization^14^. Indeed, both Ddr2 and Cyb5r3 RNAs had PDI values higher than 1, indicating that they are peripherally localized (Fig. 4B). Interestingly however, the PDI metric revealed a significant difference between the two RNAs. The Cyb5r3 RNA had a significantly higher PDI value compared to Ddr2 RNA, indicating a higher concentration at the periphery. In contrast, the DI metric was similar between Cyb5r3 and Ddr2 RNAs. Therefore, in these polarized cells, the DI and PDI metrics are not in concordance and thus reveal different aspects of subcellular RNA distributions (Fig. 4B). As mentioned above, this differentiation is expected when RNA distributions are polarized. In agreement with that, under these conditions, the Cyb5r3 RNA has a significantly higher PI index compared to Ddr2 (mean PI value 0.28 for Ddr2 and 0.51 for Cyb5r3). Therefore, the three metrics obtained through the RDI analysis can provide useful quantitative measures to describe and differentiate between cytoplasmic RNA patterns within cell populations and study their mechanistic underpinnings.

### Relationships between the three metrics can distinguish subsets of cells in a population

Furthermore, pairwise correlation of the three metrics for each RNA revealed an interesting feature. The difference between the Cyb5r3 and Ddr2 RNAs in their peripheral accumulation and polarization (detected in the graphs of Fig. 4B) did not result because the Cyb5r3 RNA has overall higher PDI and PI values in all cells of the population. Specifically, in a fraction of the cells (enclosed by the dotted line in Fig. 4C,D) the Cyb5r3 RNAs exhibit the same range of values as those exhibited by Ddr2. Apart from that population of cells, however, a distinct subset of cells (red data points in Fig. 4C) exhibit increased polarization of Cyb5r3 RNA (PI > 0.8) and increased peripheral distribution. Ddr2 RNA metrics in the same cells do not show any similar corresponding bias (red data points in Fig. 4D). This observation suggests that the difference observed between Cyb5r3 and Ddr2 RNAs is driven by the behavior of a subset of cells. Biologically, this could indicate that in this subset of cells, a particular mechanism operates that leads to specific polarized clustering of Cyb5r3 at the cell periphery. Even though in other regards the two RNAs are co-regulated (i.e. the regulation of their localization by APC), the Ddr2 RNA is not subject to this polarized clustering, at least not to the same degree as Cyb5r3 RNA. Correlating the increased polarization of Cyb5r3 RNA with activities or distributions of candidate factors could provide the basis for testable hypotheses. Thus, these results highlight how obtaining quantitative information of RNA distributions from multiple cells in a population can also serve to discern patterns observed in subsets of cells, and thus point towards biologically relevant insights.

### Effect of noise on RDI-calculated metrics

**Figure 5:**
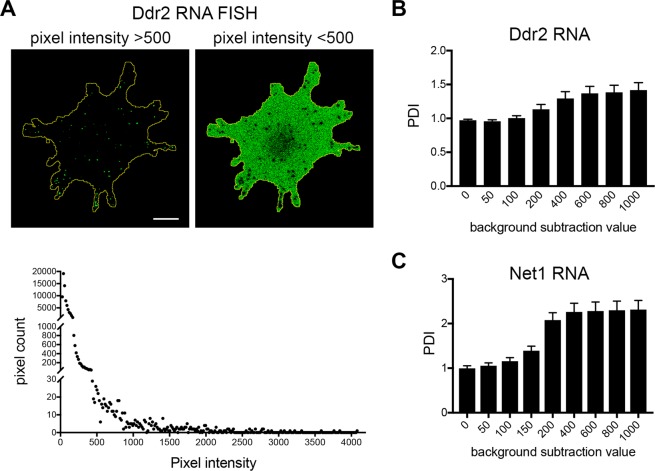
Effect of noise on RDI-calculated metrics. **(A**) Image of a cell stained to detect the Ddr2 RNA. In a 12-bit image (intensity range 0–4095), pixels with intensities more than 500, which reflect real, amplified signal (left panel), make up a small proportion of pixels within the analyzed cell area (bottom graph). By contrast, pixels with intensities less than 500, which reflect mostly background noise (right panel), make up the majority of analyzed pixels (bottom graph). Scale bar: 15 μm. (**B**,**C**) FISH images of Ddr2 (**B**) or Net1 RNA (**C**) were analyzed with the RDI calculator applying increasing values for background subtraction (n = 21). Error bars: standard error. Note that insufficient noise removal leads to PDI values close to 1 due to the preponderance of noise pixels. Increasing the background threshold leads to a gradual increase in PDI values which eventually plateau. Background thresholds recommended for analysis differ for the two RNAs (ca. 600 for Ddr2 and 400 for Net1). Difference could be due to variations of the probes used or due to variations of the microscope system used for imaging (Zeiss 780 (**C**) and Leica SP8 (**D**)).

We discuss below certain considerations that should be heeded in order to obtain RDI values that accurately reflect the distributions of the RNAs being analyzed. Given that the reported values are calculated from intensity distributions within the cell of interest, it is important to exclude from these calculations any signal originating from background noise. This is especially relevant in cases of low-abundance RNAs. As displayed in Fig. 5A, when detecting RNA species with only few copies per cell, only a small proportion of pixels report specific signal (Fig. 5A, left panel). The vast majority of pixels correspond to background noise, which usually is uniformly present throughout the cell or exhibits a perinuclear bias (Fig. 5A, right panel and graph). Even though this background signal is lower in intensity, it cumulatively affects the calculated metrics towards values that would describe more perinuclear or diffuse RNAs. For example, in the case of the peripherally localized RNA Ddr2, applying no background subtraction returns PDI values close to 1, indicative of a diffuse distribution (Fig. 5B). Increasing the background threshold leads to a gradual increase in PDI values which eventually reach a plateau at values that more accurately reflect the distribution of the RNA being analyzed (Fig. 5B). Such a response curve can provide the basis for assessing the appropriate degree of noise subtraction. Given that this value can vary depending on the specificity of probes used, the acquisition settings and detector properties (compare Fig. 5B,C), the background value to be subtracted is requested by the program as a user-defined input and can be separately specified for individual channels. In our experience, *in situ* hybridization protocols using Z-probes for signal amplification lead to good signal-to-noise. If hybridization and image acquisition conditions are maintained, then subtracting a constant amount, usually 5–15% off the lower end of the dynamic range, is sufficient for most cases.

### Consideration of 3D cellular geometry for interpretation of RDI-calculated metrics

Analysis of simulated images (Fig. 2) predicts certain values for the calculated indexes. For example, a completely diffuse RNA would have a PDI and DI index of 1. This is true in the case of 2-dimensional simulations. However, when cellular material is being observed, two-dimensional images are generated from signal originating from a three-dimensional volume. Importantly, as detailed in this section, variations in 3D morphology impact on the values of the calculated metrics and should be considered for correct interpretation of derived values.

**Figure 6:**
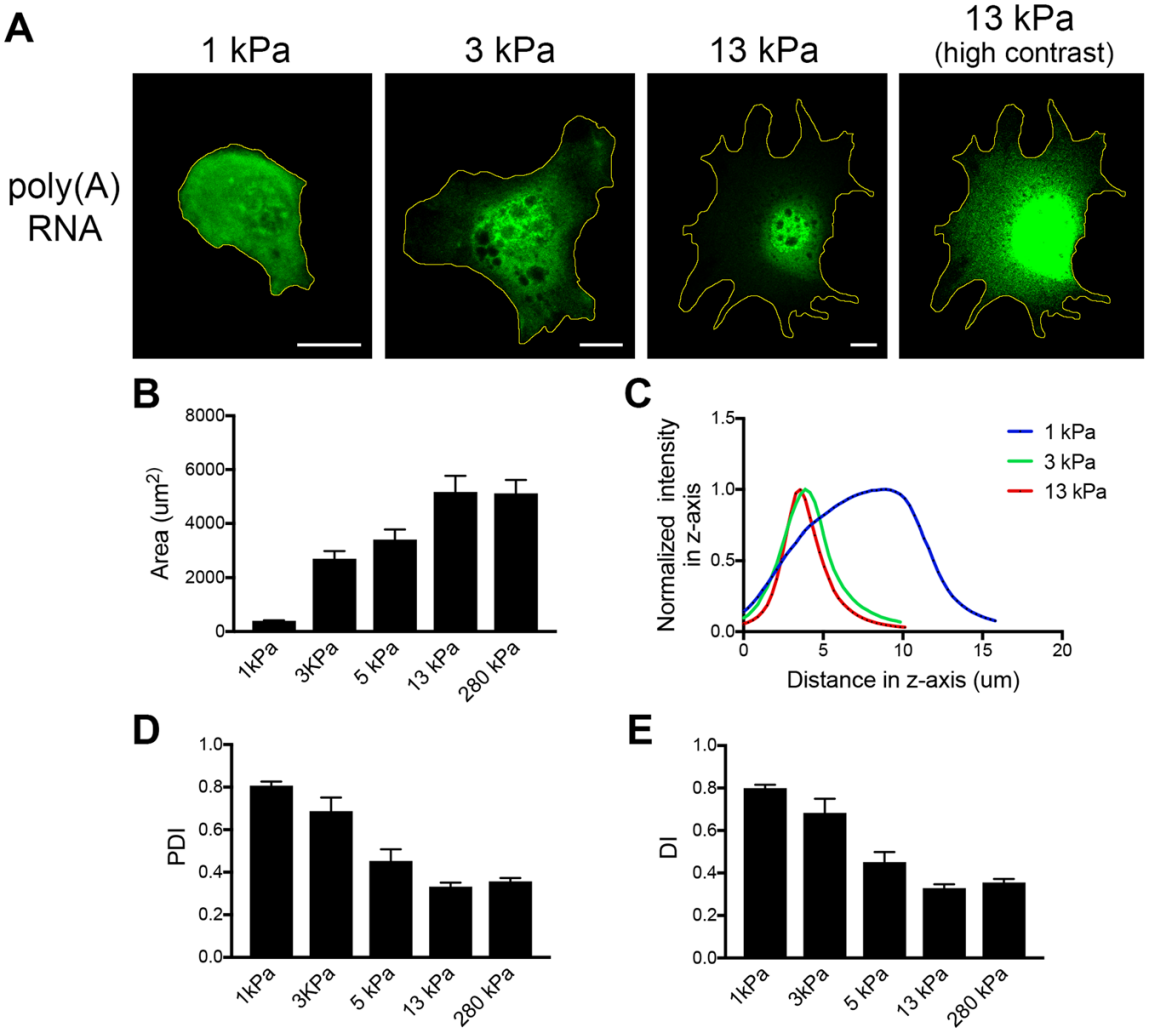
3D cellular geometry affects the values of RDI metrics. **(A**) Cells were plated on fibronectin-coated polyacrylamide gel substrates of increasing stiffness (1–13 kPa) and stained to detect polyadenylated RNA. Z-projected images are shown. Scale bars: 10 μm. (**B**) Images were analyzed using the RDI calculator to calculate RNA distribution metrics and area values (n = 11–13 cells for each condition). Cells on softer substrates (1 kPa) have a smaller area and an elongated cell body on the z-axis (**C**) indicated by the broad intensity peak along the z-axis of an image stack. Cells on substrates of increasing stiffness have increasingly larger areas and gradually reduced cell height (green and red lines in **C**). (**D**,**E)** PDI and DI metrics of polyA RNA signal from cells analyzed in B. Error bars: standard error. Note that increasing stiffness and area correlate with decreased PDI and DI values of polyA RNA, reflecting changes in the spatial distribution of the bulk cytoplasmic volume.

To image different 3D morphologies of the same cell population, we plated fibroblast cells on fibronectin-coated polyacrylamide substrates of varying degrees of stiffness. On soft substrates (1 kPa Young’s modulus) cells remain mostly round and don’t spread efficiently (Fig. 6A). They are thus characterized by a small spreading area (Fig. 6B) and an increased height, evident by a broad intensity peak along the z-imaging axis (Fig. 6C). On substrates of increasing stiffness (3, 5, 13 and 280 kPa) cells spread gradually more (Fig. 6A,B) and the cell height is reduced, seen by a narrower intensity peak along the z-axis (Fig. 6C). On a 2-dimensional z-projected image, cells exhibiting different degrees of spreading, will have a quite distinct distribution of the bulk cytoplasm. On less spread cells, cytoplasmic material will appear uniform throughout the cell body. By contrast, in highly spread cells, the cytoplasm will appear unevenly distributed in central versus peripheral regions (with higher cytoplasmic material around the nucleus and a gradual decrease towards the periphery with a thin layer of cytoplasm reaching into the mostly membranous protrusions). Consequently, a molecule that is freely diffusing in the cytoplasmic volume would appear more perinuclear in a projected image of a spread cell and the signal would have a PDI or DI index less than 1.

To assess these predictions, we plated cells on substrates of varying stiffness and detected polyadenylated RNA (through oligo-dT hybridization) as a surrogate of the cytoplasmic volume accessible to RNA molecules (Fig. 6A). Indeed, consistent with the above expectations, in 2D-projected images, polyA RNA appears uniformly diffuse in the cytoplasm of less spread cells (PDI and DI values close to 1) but appears gradually more perinuclear (evident by decreasing PDI and DI values) as the spreading area of the cells increases (Fig. 6D,E). This doesn’t mean that polyA RNAs are actively excluded from protrusions, but rather reflects the changes in the spatial distribution of the cytoplasmic volume. Therefore, while the 2D simulations provide some reference points, the meaning of the actual reported values should be interpreted in the context of the 3D geometry of the cells being observed. We suggest that the distribution of polyadenylated RNA would be a useful internal control against which distributions of individual RNAs could be compared and interpreted.

## Discussion

We report here an automated, interactive analysis method for the rapid calculation of metrics that quantitatively describe RNA distributions in cell populations. This Matlab-based program allows the automated identification of cell boundaries thus reducing the amount of time required for analysis of a series of images. As an additional step to increase accuracy, intermediate steps in the analysis are presented to the user for validation to avoid errors in the final output. Overall, these features will facilitate the rapid analysis of large datasets that can better reflect the overall cell populations being examined. They will also allow for a more confident assessment of the uniformity exhibited within a sampled population, or of the existence of potentially interesting behaviors manifested in subsets of cells.

We have also highlighted considerations that should be taken into account when interpreting these metrics and for comparative analyses. Given sufficient elimination of background noise, the metrics calculated with this program can robustly compare distributions of different RNAs within the same cells. To signify that, the script labels one RNA species as ‘localized’ and the other as ‘control’. The identity of the RNA that can serve as an appropriate control can vary depending on the cell type, the particular conditions and the goal of the experiment. We suggest that a useful comparison is against the distribution of the general polyadenylated RNA population in the cytoplasm of the cells being analyzed. This can be detected through oligo-dT hybridization and can provide a measure of the overall RNA distribution against which specific RNAs can be compared.

Apart from comparisons of different RNAs within the same cells, understanding the mechanisms underlying localization of individual RNAs to particular compartments requires comparison of RNA distributions across various experimental conditions. It is important to note that in these types of experiments, any observed changes in RDI metrics could result either from perturbation of cellular mechanisms acting specifically on the RNA of interest or they could result indirectly as a consequence of changes in 3D cellular morphology, which, as detailed in the results presented above, can affect the calculated values. To provide a means of assessing changes in 3D cellular morphology among cell populations, the program reports the cellular area of each observed cell. For cells of similar volume, changes of their 2D footprint would be indicative of potential changes in 3D geometry. We emphasize however that area values are just indicative and cannot support by themselves conclusive inferences regarding 3D architecture. Imaging the distribution of polyadenylated RNA could provide a more direct indication of whether any observed differences are specific to the RNA of interest or whether they result from changes in the relative distribution of the bulk cytoplasm within the cells being observed. An alternative approach could involve the use of substrates of defined size and shape^16^. Such micropatterned substrates can be used to confine cells within a particular shape and thus circumvent the confounding effects brought about by drastically different morphologies.

We note that while we have implemented this program for the study of RNA distributions, the same analysis and metrics can be used to quantitatively assess the cellular distribution of any molecule or activity detected through microscopy-based imaging.

## Supplementary information

RDI Calculator_User Manual

Dataset 1

## Data Availability

The datasets generated and analyzed in the current study are available from the corresponding author on reasonable request.
