# Supplementary material for: RDI Calculator: An Analysis Tool to Assess RNA Distributions in Cells
Source: Sci Rep. 2019 Jun 4;9:8267. doi: 10.1038/s41598-019-44783-2 (PMC6547641; doi:10.1038/s41598-019-44783-2)
Supplement: Supplementary file 1 — RDI Calculator_User Manual [file 41598_2019_44783_MOESM1_ESM.pdf]

RDI Calculator: An Analysis Tool to Assess RNA Distributions in Cells

Michael Stueland, Tianhong Wang, Hye Yoon Park, Stavroula Mili

Step-by-step instructions

This program is designed to take in 4-channel .lif files or folders containing .tif files. Images can be single-plane or z-stacks, and can also be any bit depth, though 12- or 16-bit is recommended. Images should have a nuclei channel, a cell mask channel, and two RNA channels. Nuclei and cell mask channels should have good contrast to background and can be saturated.

1) Add to Matlab folder all the required scripts and select 'RDI_Calculator_V1s7_LIF.m' (if analyzing a .lif file), or select 'RDI_Calculator_V1s7_TIF.m' (if analyzing a series of Tiff images). Hit the 'Run' button

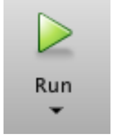

2) Next, select the .lif file …

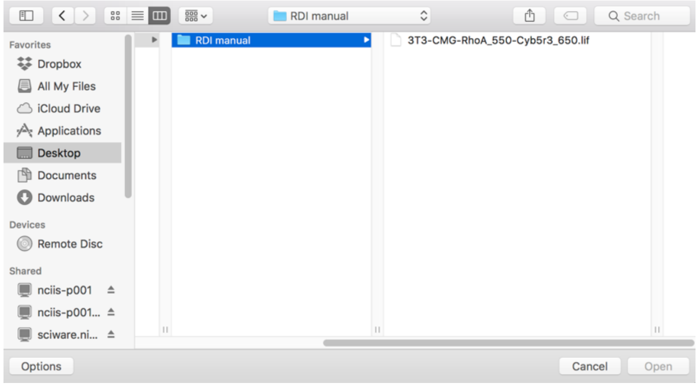

… or a folder, containing 1 or more 4-channel .tif files

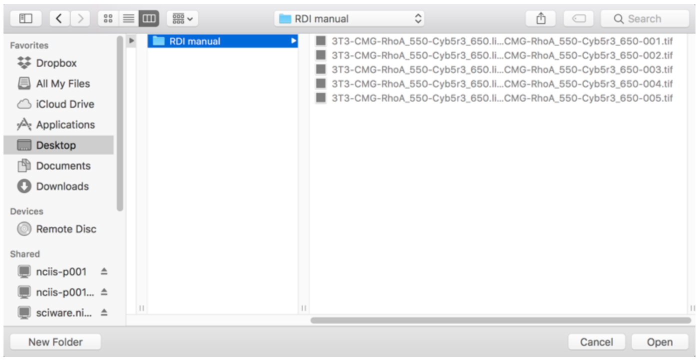

3) Input the channels in the order they are acquired, along with the bit-depth of the images for proper viewing. For the test .tiff images provided, input the following:

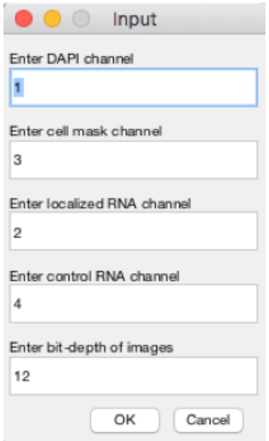

4) Enter amount to subtract from each channel. For instance, in a 12-bit image the maximum intensity is 4095. A subtraction of 300-600 from the RNA channels may be appropriate to remove any background noise. This may also be used to 'clean up' the cell mask or nuclei channels in case of high background noise. For the test images provided input the following:

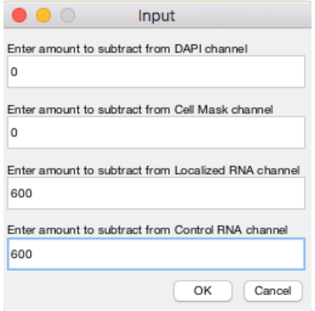

5) The program provides the option for the signal within the nuclear mask to be subtracted from the other channels in case it is desirable for some applications.

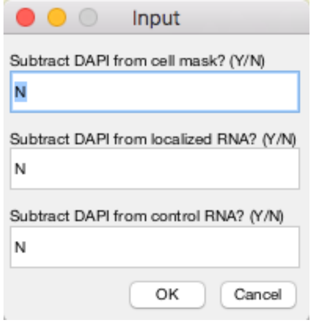

6) Next, the program asks the user if they would like to run the RDI analysis. Generally, the user will want to select 'Yes', unless the only desire is to produce saved masks for the nuclear and cytoplasmic signal.

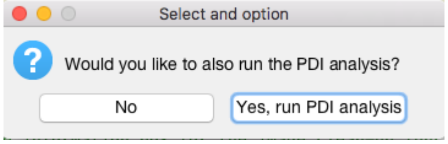

7) A reminder to the user to save images

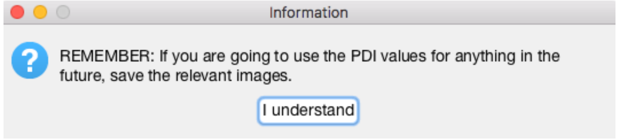

8) Next, the program will ask the user if they would like to use a set of techniques to batch-clean up their images in the cell mask channel if, for instance there is a lot of background noise. These same options are available per-image, and this is simply the batch option. For images with good signal-to-noise 'No' can be selected. The test images provided can be run through without any further processing (proceed to step 9A below). For more noisy images, select 'Yes' (proceed to step 9B below).

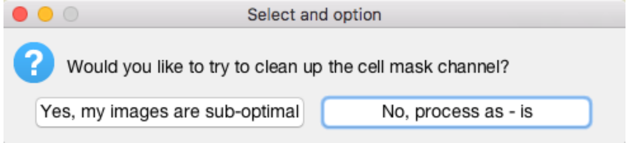

9A) For each image, the program displays an overlaid boundary for the cell mask with the RNA channels (to make sure that all RNA spots are included in the generated cell mask), the signal from the cell mask channel with an overlaid boundary of the generated cell mask and the signal from the nuclear channel with an overlaid boundary of the generated nuclear mask. Note that the program will always pick the largest (by area) object in the image to be the cell of interest, and the largest object within the cell to be the nucleus. A pop-up window is shown, giving the option to the user to first save the cell mask and then the nuclear mask. (If any one of the generated masks is not acceptable, 'No' can be selected (proceed to step 9C below)).

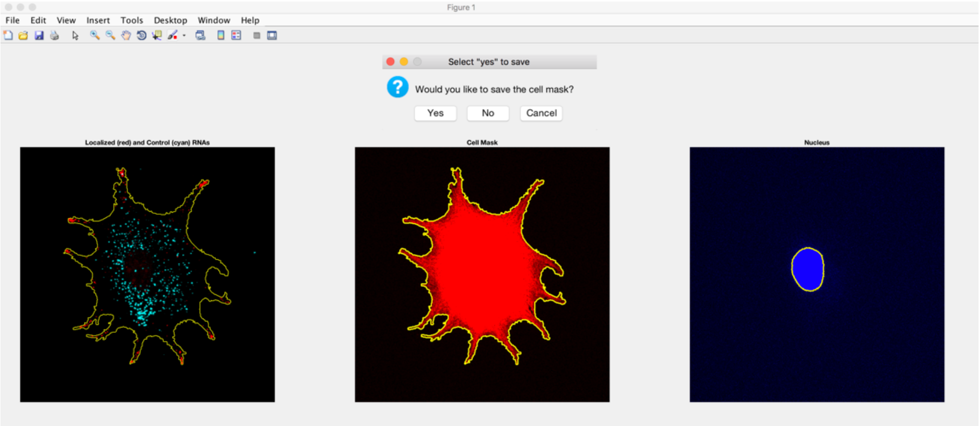

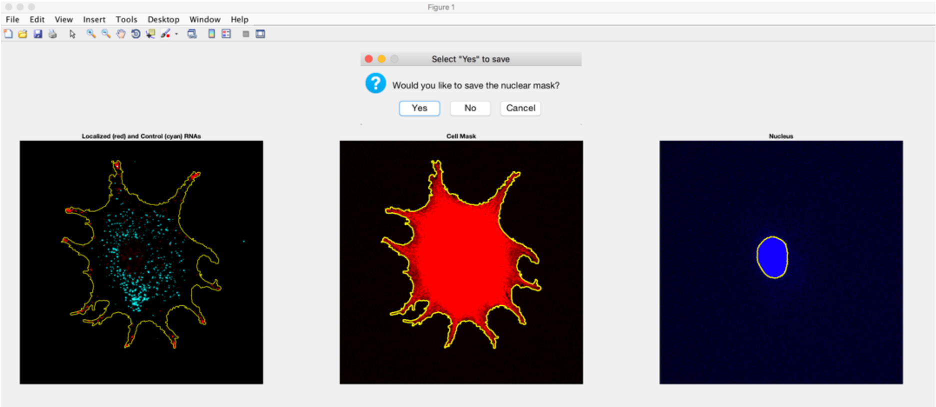

The program iterates through each image in the original directory. If the masks are accepted all relevant data are stored in the original image folder, including the masks, Z-projected images with user-determined subtraction, and a .csv file with all relevant analysis data. The analysis data (PDI, PI, DI, Cell area, and average intensity) can also be quickly obtained in the variable ‘A’ within Matlab.

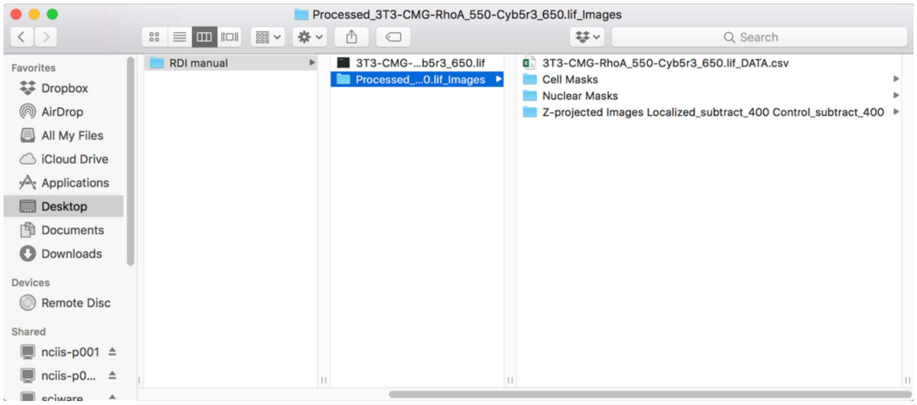

Options for cleaning up the mask channels or manually altering masks.

For noisy images, the default parameters might not successfully generate acceptable cell masks. In such a case, the user can specify in step 8 above that the 'images are sub-optimal' and attempt to alter the parameters

9B) If the user chooses the 'my images are sub-optimal' option, a small information box is presented

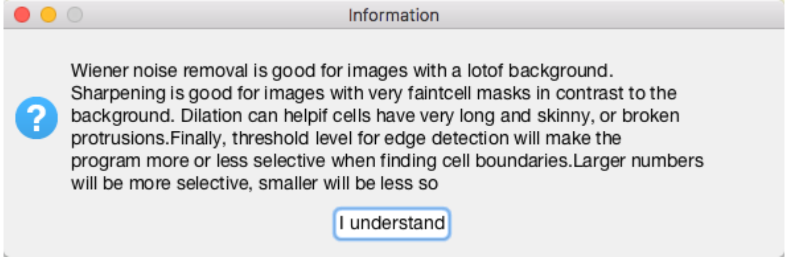

Four options are available to the user to try to clean up the cell mask channel. These methods can be performed multiple times. The first option is the application of the Wiener-filter, for noise removal. The second will sharpen the image, boosting contrast between signal and background. The third will dilate the cell mask by one pixel in all directions. The fourth will modify the threshold for the sobel edge detection.

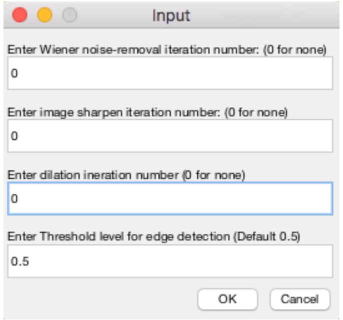

The program will use the new parameters to generate masks and, as in step 9A, it will iterate through the images, displaying the generated masks and asking the user to save or not the images.

As an example, below is a particularly poor cell mask. The brightness has been increased significantly for visibility.

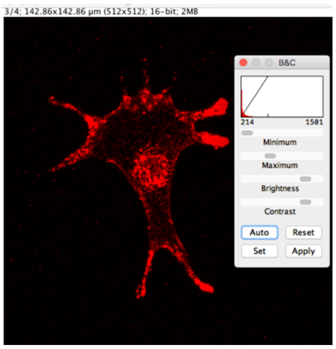

This is the automatically calculated mask.

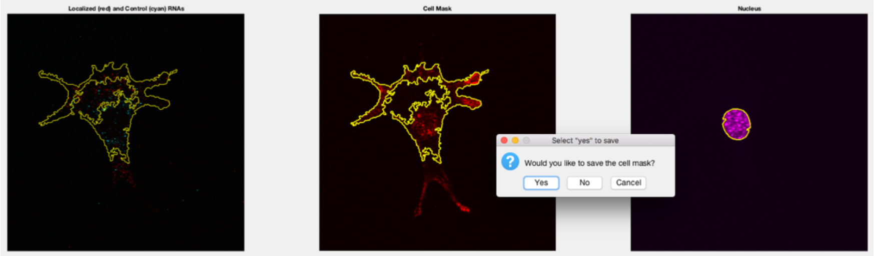

However, with the correct settings, an acceptable mask can be generated. The following settings are applied to increase sharpness, dilate the cell mask slightly, and decrease the stringency of the Sobel edge detector.

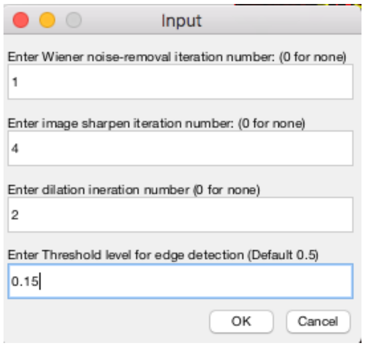

The new mask is much closer to where the ‘true’ cell boundaries are.

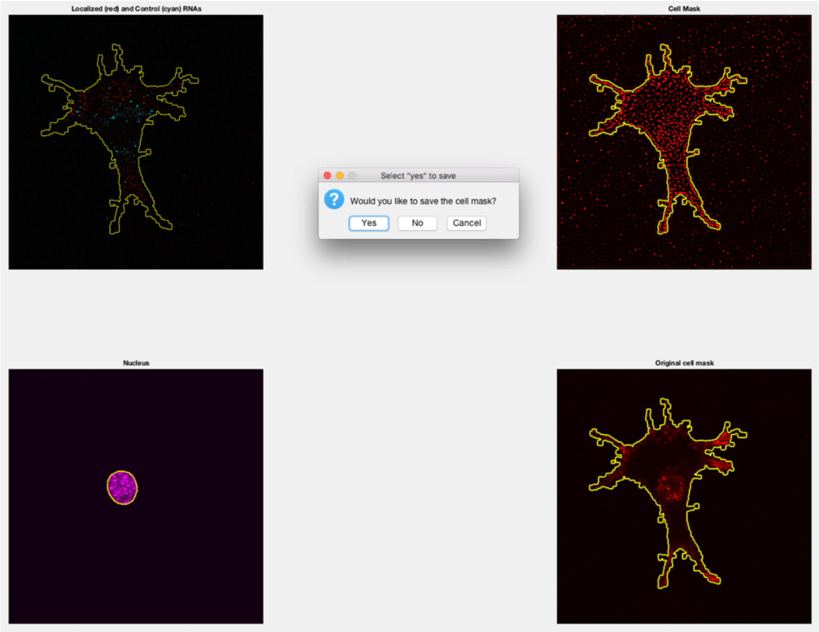

9C) If any of the individual generated masks is not acceptable, 'No' can be selected when the user is asked to save the mask. In this case the user can choose to use 'advanced options' or to 're-draw' the mask.

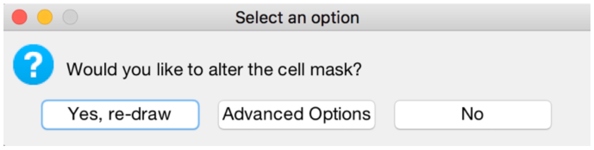

'**<u>Advanced options</u>**' offer the ability to change the same parameters as described in step 9B, but these will now be applied only to the individual image being analyzed.

The '**re-draw**' option offers different ways that the user can manually change the mask.

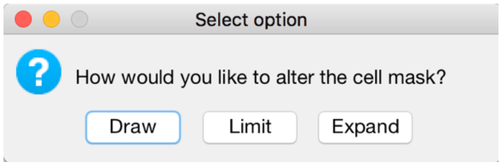

The '**<u>Draw</u>**' option allows the user to use a polygon to draw a mask of choice. Once the option is selected, the image on the right will change to a display of the cell mask in green and the nuclei in magenta (overlap appearing white). A small pop-up box will also appear, giving basic instructions. Click 'OK' to begin drawing within the right window.

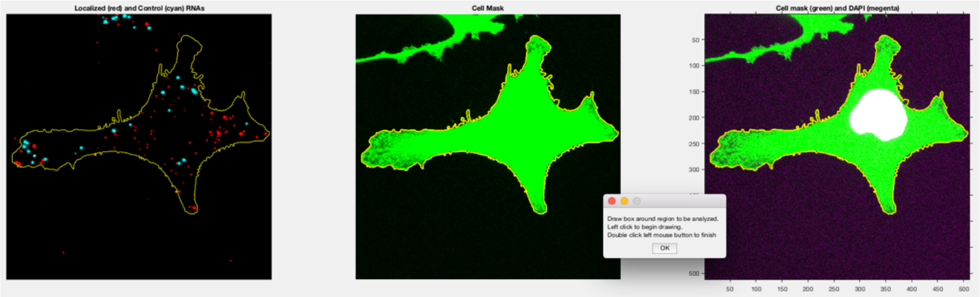

In the '**<u>Expand</u>**' option, the user draws a polygon connecting to the calculated cell mask boundaries. Double click to close the polygon.

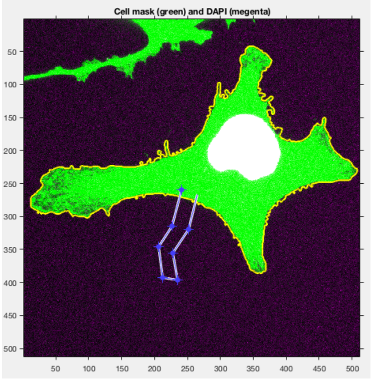

The program will then display the modified cell mask to the user. This option is useful if specific areas have not been included in the original cell mask.

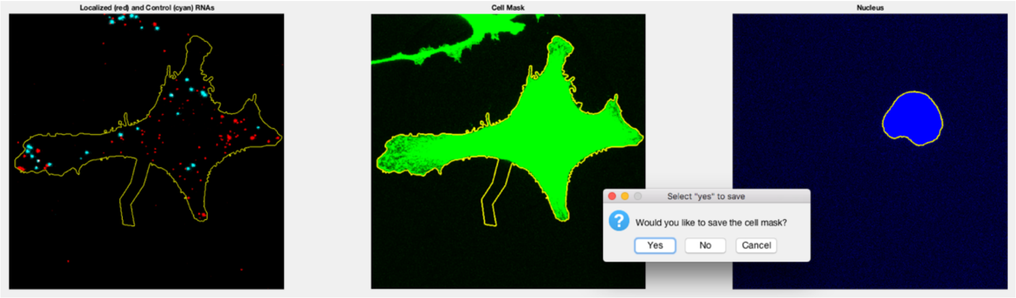

In the '**<u>Limit</u>**' option, a broad polygon can be drawn around only the area the user would like to be included in the analysis.

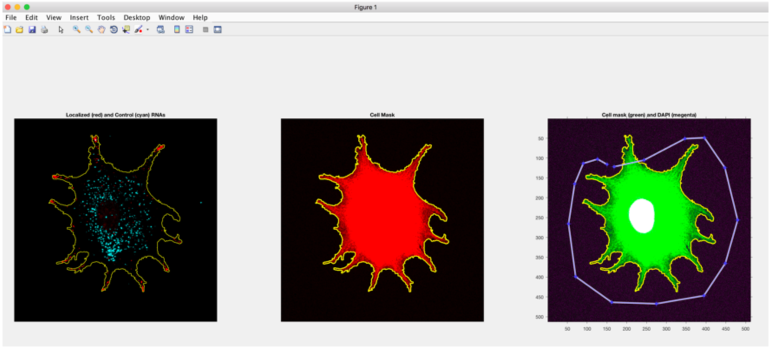

The resulting mask will exclude any areas not included in the polygon. This can be useful if the cell of interest is in contact with other cells, which thus become part of the original mask and need to be excluded.

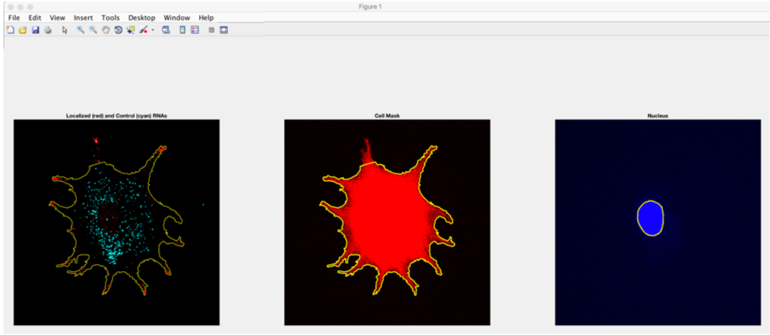

The user can make as many edits like these as necessary. If they again click 'No' to saving, the user can return to the drawing options.

Note 1: The 'advanced options' are unavailable after the user has altered the mask with the polygon draw tool. If needed, masks should first be optimized with the 'advanced options', then small changes made with the polygon 'draw' tool.
Note 2: The nuclear mask is always identified within the area determined by the cell mask.
Note 3: Re-drawing options can also be used to alter the nuclear masks.
