# Supplementary figures and images for: RDI Calculator: An Analysis Tool to Assess RNA Distributions in Cells
Source: Sci Rep. 2019 Jun 4;9:8267. doi: 10.1038/s41598-019-44783-2 (PMC6547641; doi:10.1038/s41598-019-44783-2)

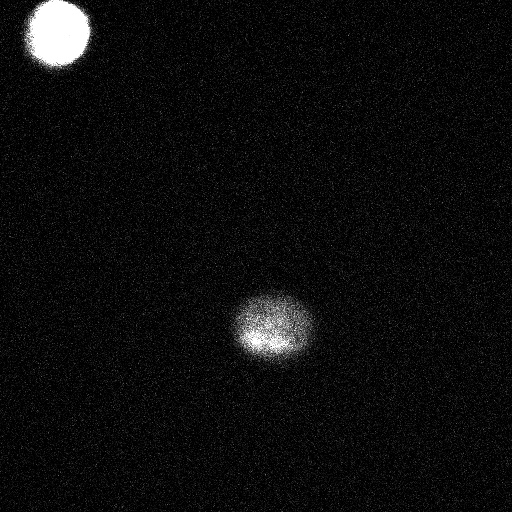

Supplement: Supplementary file 2 — Dataset 1 [file 41598_2019_44783_MOESM2_ESM.zip › RDI Calculator-test images/3T3-CMG-RhoA_550-Cyb5r3_650.lif - 3T3-CMG-RhoA_550-Cyb5r3_650-002.tif]

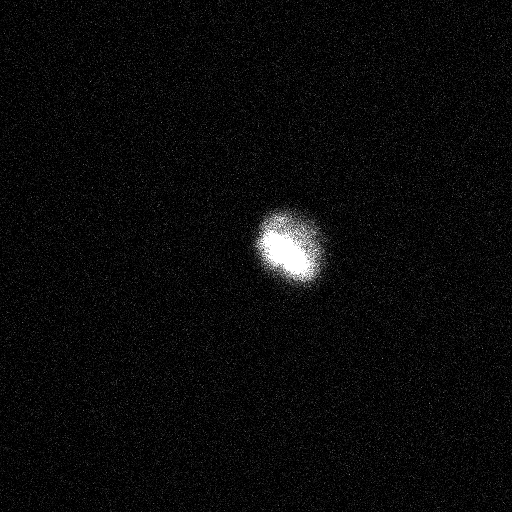

Supplement: Supplementary file 2 — Dataset 1 [file 41598_2019_44783_MOESM2_ESM.zip › RDI Calculator-test images/3T3-CMG-RhoA_550-Cyb5r3_650.lif - 3T3-CMG-RhoA_550-Cyb5r3_650-003.tif]

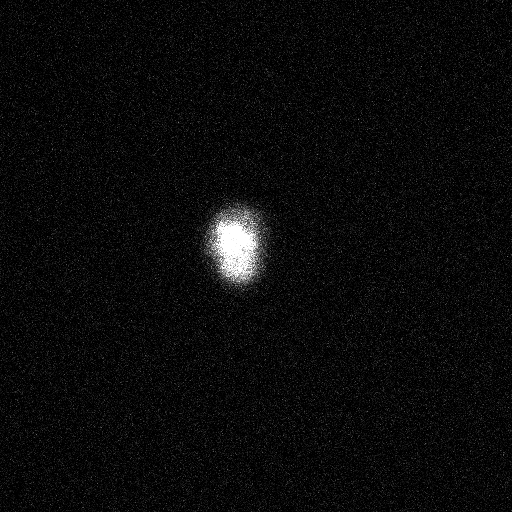

Supplement: Supplementary file 2 — Dataset 1 [file 41598_2019_44783_MOESM2_ESM.zip › RDI Calculator-test images/3T3-CMG-RhoA_550-Cyb5r3_650.lif - 3T3-CMG-RhoA_550-Cyb5r3_650-001.tif]
